# Microscopic Exploration of Water Permeation and Ion Rejection for Edge Amine-Functionalized GO Nanoslits

**DOI:** 10.3390/membranes15110334

**Published:** 2025-11-04

**Authors:** Yinfeng Pei, Wenjin Li, Xiaoning Yang

**Affiliations:** State Key Laboratory of Materials-Oriented Chemical Engineering, College of Chemical Engineering, Nanjing Tech University, Nanjing 211816, China

**Keywords:** layered GO membranes, desalination, edge functionalization, nanoslits, molecular simulation

## Abstract

Layered graphene oxide (GO) has emerged as an ideal membrane structure for water desalination. In GO-stacked structures, the slit gaps between GO nanosheets can serve as critical pathways for molecule permeation. Exploring the permeation mechanisms of functionalized GO nanoslits is critical for improving the separation performance. Herein, molecular simulations were performed to investigate the water permeation and ion rejection for six types of ionic solutions by considering edge-amino functionalized GO (NGO) slit membranes. The NGO slit exhibits higher ion retention while maintaining reasonable water permeability. Edge amine groups can interact strongly with water molecules and immobilize ions, thus enhancing ion rejection. The thermodynamic free energy for ion passing was simulated to explain the unique ion rejection mechanism of amine-functionalized GO slits. The thermodynamic barrier for ion rejection can be considered as the delicate combination of the ion dehydration effect and the slit-generated attraction. The ion dehydration accounts for a repulsive contribution, which is the controlling portion in governing the free-energy profile. Overall, our work is important and valuable for the development and design of new-type layered GO membranes.

## 1. Introduction

As global water scarcity intensifies, the application of new technologies for seawater desalination and wastewater treatment is becoming increasingly important [1,2,3]. In this aspect, membrane separation technology has attracted significant interest for its high efficiency, low energy consumption, and environmental advantages. Using new membrane materials is essential for advancing the capabilities of membrane separation technologies [4,5].

Currently, two-dimensional (2-D) materials have been widely applied in the field of membrane separation [6,7]. Layered graphene oxide (GO) is considered an ideal 2-D membrane structure for water treatment and desalination, attributed to its exceptional water permeability, favorable physicochemical properties, and ease of large-scale fabrication [8,9,10]. The laminated structure formed by the stacking of GO nanosheets creates unique nanochannels and nanoscale edge slits for efficient molecular permeation [11,12]. Particularly the slit gaps formed between edge-to-edge nanosheets are widely present in stacked multilayer GO membranes, thereby serving as the critical pathways for molecular or ion permeation [13] and providing important desalination function [14]. Therefore, the exploration and design of GO nanoslits are very critical for improving the desalination performance of GO layered membranes.

Previous work has shown that slit edge pattern and slit spacing are the main factors affecting the desalination performance of 2-D slit membranes [15,16,17]. Currently, various approaches have been proposed to functionalize or modify the slit edge structures [18,19,20]. Among these methods, chemical modification has proven to be an effective approach for improving ion rejection without significantly compromising water permeability [21,22]. It has also been reported [23] that different edge functional groups of GO membranes can achieve high selectivity for ions. These results might demonstrate the possibility of tuning water and ion permeation by functionalizing the GO edge as an effective strategy.

Amine groups are considered to be excellent units for the chemical modification of carbon materials. The introduction of amine functional groups could affect the desalination performance of GO membranes [24,25]. Qian et al. [24] proposed a plasma scheme to develop functionalized GO membranes (FGOM) enriched with amine functional groups on GO surfaces. They found that the amine functional groups of FGOM exhibit strong electrostatic interactions with ions, resulting in a high monovalent/divalent cation selectivity while achieving ultra-high water flux. Additionally, amine modification prevents the stacking of graphene sheets and enhances the physical and chemical robustness of the membrane [26,27]. A recent study [28] has confirmed that a reduced GO sheet possesses numerous vacancy/defect sites, especially at the edges, providing ample positions for the attachment of -NH_2_ groups. The above experimental studies provide background support for realizing amine-functionalized slit edges in GO layered membranes. Therefore, it is hypothesized that such edge-functionalized amine GO membranes will improve the desalination performance of GO membranes. However, current conventional GO membranes are mainly functionalized with the edge carboxyl groups. As far as we know, there has been no comprehensive study on the desalination mechanisms and performances of GO nanoslit membranes modified with amine groups.

In this work, using molecular dynamics (MD) simulations, for the first time, we simulated and compared the water permeation and ion rejection capabilities for six different aqueous ion solutions (K⁺, Na⁺, Ca^2^⁺, Mg^2^⁺, Al^3^⁺, Ga^3^⁺) by using edge functionalization GO (NGO) slit membranes with neutral amine groups. These metal ions are commonly present in seawater and wastewater treatment processes, and their excessive accumulation can cause serious risks to human health and the environment [29,30]. We observed that the edge amine-functionalized GO slits could exhibit higher ion rejection for metal ions. Additionally, we simulated the potentials of mean forces (PMFs) of ions passing through the NGO nanoslits, providing molecular insight into the ion rejection mechanisms, which is expected to improve the understanding of nanoscale molecular permeation. Our findings contribute to the further design and development of nanomaterial GO-layered membranes for seawater desalination and wastewater treatment.

## 2. Simulation Models and Methods

### 2.1. Simulation Models

**Figure 1:**
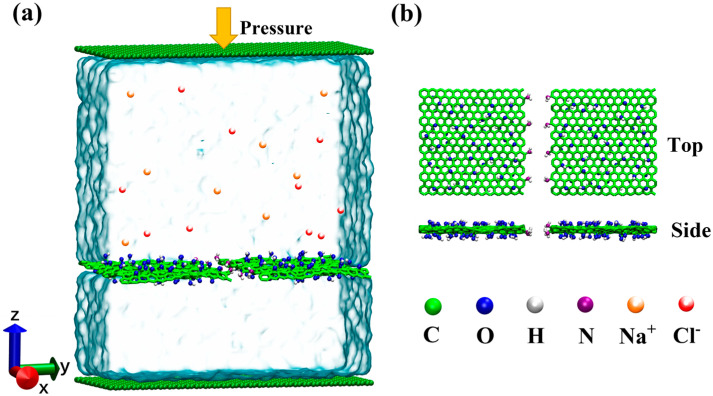
(**a**) A schematic of the simulation system, consisting of the NGO nanoslit membrane and two chambers on both sides of the membrane. The upper chamber contains an aqueous ionic solution and is equipped with a graphene piston, while the lower chamber includes pure water with another graphene sheet. (**b**) NGO nanoslit structures, including top and side views. The atom color codes are white for hydrogen, blue for oxygen, green for carbon, purple for nitrogen, orange for Na^+^, and red for Cl^−^.

In this study, we utilized non-equilibrium MD simulation methods to simulate the pressure-driven desalination process [31]. As illustrated in Figure 1a, the simulation system consists of a slit membrane formed by the NGO sheets with the xy-dimensions being 30.0 × 30.0 Å. Generally, the surface of each GO sheet was enriched with epoxy and hydroxyl groups at a total oxidation degree of 20%. In Figure 1b, for the NGO GO sheet, four amine (-NH_2_) groups were attached to the GO edge. Herein, following the previous works [9,32,33], the unsaturated edge carbon atoms were not passivated in our classical MD simulation. In the simulation process, nanoscale slit gaps are typically generated by altering the alignment of two GO nanosheets, creating slit widths from 8 to 12 Å, defined as the distance between the carbon atoms at the edges of the two GO sheets. This is expected to explore the impact of geometric parameters on desalination performance [34]. The ionic solution and pure water were positioned on the upper and lower sides of the GO slit membrane, respectively, defined as the feed side and the permeate side. In all simulation setups, the lengths of the permeation and feed sides along the z-direction were 31 Å and 59 Å, respectively. Additionally, a 100 Å vacuum region was positioned below the permeate side along the z-direction. The 0.5 M concentrations of NaCl, KCl, MgCl_2_, CaCl_2_, AlCl_3_, and GaCl_3_ solutions were used as the feed solutions.

### 2.2. Force Field and Simulation Details

All MD simulations were performed using the LAMMPS (18 August 2022) software package [35]. To ensure the structural stability of the GO membrane model, the center of mass of the skeletal carbon atoms within the GO plane was held fixed, while the functional groups on the surfaces and edges were allowed to be flexible. We used the Tersoff–Brenner (T-B) potential [36] to describe the effective many-body interactions between carbon atoms in the GO basal plane, which has been commonly and successfully used to describe carbon-based materials [37,38]. The all-atom optimized potential for liquid simulations (OPLS-AA) force field [39,40,41] was employed to simulate the functional group atoms, which has been extensively applied in previous simulation studies of desalination using layered GO membranes [9,32,42]. The SPC/E model [43] was used to model the water molecules. The ionic force field parameters were sourced from the literature [44,45,46,47,48], which has been used in simulating ion permeation [9,30,31]. Generally, the Lorentz–Berthelot mixing rules are used to handle the Lennard–Jones (L-J) interactions between different particles. For the carbon atoms in the graphene framework and the oxygen atoms in water molecules, specific L-J parameters (ε = 0.392 kJ mol^−1^, σ = 3.19 Å) were applied, which can represent experimental water contact angles on graphite surfaces [49]. All the force field parameters used in the simulations are listed in Tables S1 and S2. A cutoff radius of 10 Å was selected for L-J interactions between particles, and the particle-particle-particle-mesh (PPPM) method was used to calculate long-range electrostatic interactions with a tolerance of 10^−5^ [50]. Periodic boundary conditions were applied in the x and y directions, while the non-periodic condition was used along the z-direction.

Throughout the simulation process, the system was maintained under the NVT ensemble, using a Nosé–Hoover thermostat to maintain the temperature at 300 K. During the pressure-driven permeation simulations, two vertically movable rigid graphene nanoplates were used as pistons, and pressure was applied along the z-axis. High pressures (100, 200, 300, and 400 MPa) were applied to the piston positioned above the feed side, while an atmospheric pressure of 0.1 MPa was applied to the piston below the permeate side. The carbon atoms in the pistons were modeled as uncharged L-J spheres [41,51]. To generate the desired pressure differential (∆P), a force (f) was applied to each carbon atom in the pistons, calculated as f=∆P·A/n [52], where A represents the area of the piston plate, and n is the total number of atoms in the graphene piston plate, with ∆P being the pressure applied during the simulation. Higher applied pressures helped decrease thermal noise and enhance the signal-to-noise ratio within nanosecond time scales [53,54]. The timestep for the overall simulation process was set at 1 fs, and each system was simulated for at least 20 ns to ensure sufficient statistical data and reliable simulation outcomes.

## 3. Results and Discussion

**Figure 2:**
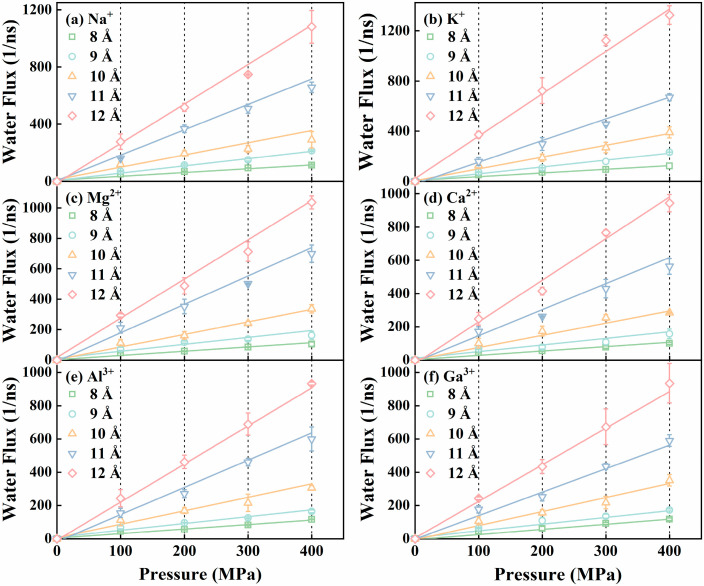
Water fluxes as a function of the applied pressure difference for the six different ionic aqueous solutions through the NGO nanoslits with varying slit spacing. (**a**) Na^+^; (**b**) K^+^; (**c**) Mg^2+^; (**d**) Ca^2+^; (**e**) Al^3+^; (**f**) Ga^3+^.

First, we simulated the water permeation behavior for six kinds of ionic aqueous solutions passing through the GO slit membrane models. The change in the water permeation number with the simulation time was found to display a linear behavior, demonstrating a stable permeation process [55]. Then, we extracted the water flux (in units of 1/ns) based on the slope of the time-dependent water permeation curves. Figure 2 shows the linear increase in the water number fluxes versus the pressures, ranging from 100 to 400 MPa, in the NGO slit membranes. The linear correlation coefficients of determination (R^2^) are all greater than 0.99. Although the simulated pressures are significantly higher than those typically used in actual desalination processes, the linear relationship between the flux and pressure suggests the permeation performance can be extrapolated to the lower operating pressures (5–8 MPa) in actual applications [56,57].

**Figure 3:**
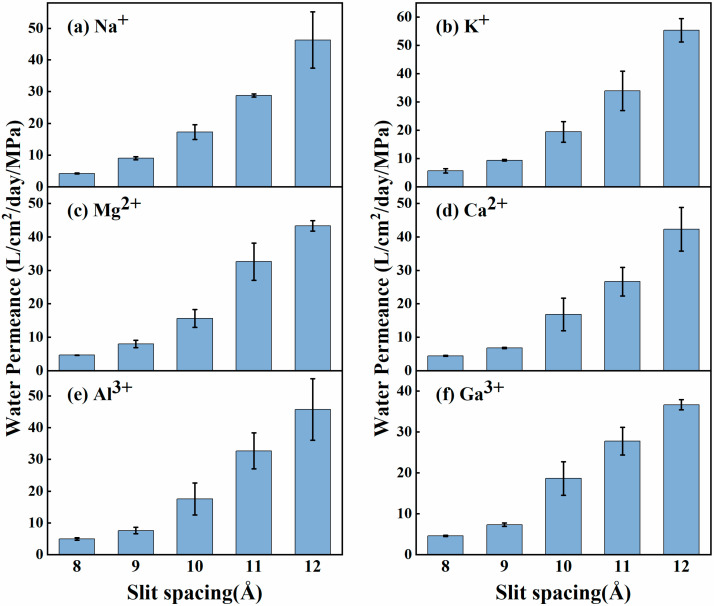
Water permeability variations in six different metal ionic aqueous solutions through NGO nanoslits with the spacing ranging from 8 Å to 12 Å. (**a**) Na^+^; (**b**) K^+^; (**c**) Mg^2+^; (**d**) Ca^2+^; (**e**) Al^3+^; (**f**) Ga^3+^.

According to the above data, we further evaluated the water permeability through the functionalized GO slit membranes (in units of L/cm^2^∙day^−1^∙MPa^−1^), defined as the water-passing volume per unit crossing membrane area per unit time per unit pressure [55]. Herein, the slit porosity of 10% was used on the cross-section surface of the GO slits in the permeability calculation. Figure 3 presents the water permeabilities for the NGO membranes across different slit widths. As expected, with the slit width increasing, the simulated permeability of monolayer NGO slit membranes generally shows a growing trend and is larger than the experimental data of the 2-D layered MXene membranes [58]. At the same time, we also compared our results with simulation studies of other slit materials. Although it is relatively difficult to make a rigorous comparison of the simulation results for different types of slit structures, the water permeabilities of the slit GO membranes are within the range of 5–60 L/cm^2^∙day^−1^∙MPa^−1^, which is overall comparable to other types of slit membranes [59,60]. In addition, there exists a certain difference in the water permeability for various ionic solutions. That is, a high-valent ion solution generally exhibits a lower water permeability, implying that the ion with a high valence has a certain impedance on the water permeation.

**Figure 4:**
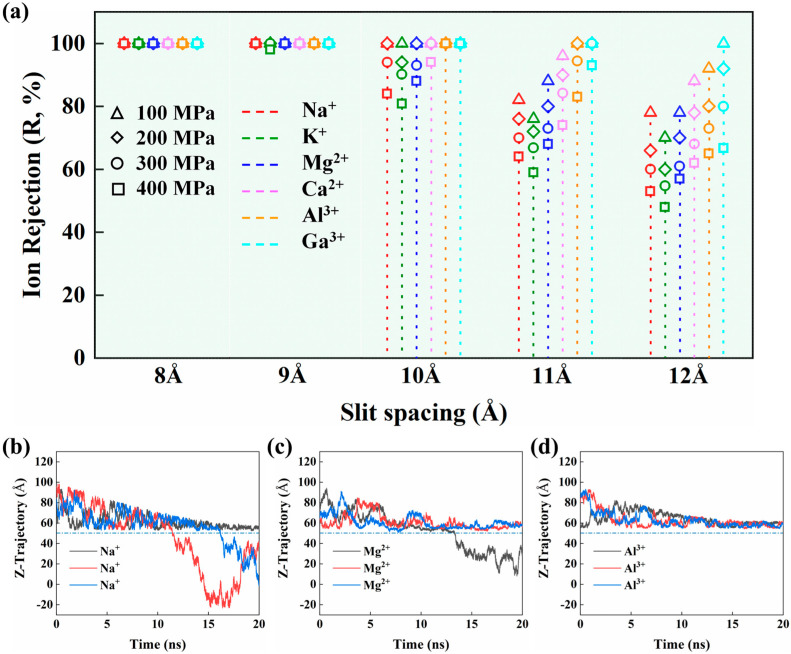
(**a**) Variations in the average salt rejections (*R*) of six metal ions with slit widths and pressures for NGO slits. (**b**–**d**) Moving trajectories of randomly selected three Na^+^, Mg^2+^, and Al^3+^ ions along the z-direction as a function of simulation time at 300 MPa and a slit gap of 10 Å. The blue dashed lines at 50 Å indicate the positions of the NGO membrane.

Figure 4a shows the cation retention behavior across the NGO slit membranes with various widths and pressures. The ion rejection rate (*R*) was calculated as *R* = 1 − *N*_1/2_/*N*_0_ [61], where *N*_0_ denotes the initial number of ions on the feed side and *N*_1/2_ represents the number of ions on the permeate side after half of the water molecules have traversed the slit. In the computation, if the center of mass (COM) of an ion is below the position of the NGO membrane in the z-direction, it is considered that the ion has passed through the slit. As expected, the ion retention rate decreases with the slit width and pressure increasing. However, it is noted that the amine-functionalized GO slits can achieve 100% of the ion retention rates for all the cations under the pressure of 100 MPa below the slit width of 10 Å. The ion rejection of the amine-functionalized GO membrane is generally higher compared to other types of 2-D slit membranes [60,62]. The performance suggests a high ion rejection function of the edge amine-functionalized GO slits. Furthermore, the high-valence ions, such as Al^3+^ and Ga^3+^, exhibit significantly higher rejection rates in the NGO slit membranes, as compared to lower-valence ions. This behavior could be attributed to the stronger electrostatic interactions of the high-valence ions.

Overall, the NGO slit membranes can exhibit high retention rates for metal ions. To provide a more visual understanding of ion rejection behavior within the slit membranes, Figure 4b–d show ion permeation movement trajectories for three typical Na^+^, Mg^2+^, and Al^3+^ ions along the z-direction for the NGO slit membranes. It can be observed that trivalent ions usually have a greater degree of difficulty when passing through the nanoscale slits during the simulation period, further corroborating the strong metal ion retention action from the GO slit edges.

To understand the effects of edge functionalization and ion types on the water permeation, the simulated water permeability through GO slit membranes was fitted by the improved Stokes model [62],

(1) $$\frac{Q_{v}}{∆P}=\frac{\pi L{d_{eff}}^{2}}{32\mu }+\alpha d_{eff}$$

where *Q_v_*, in unit of nm^3^/s, represents the volumetric flow rate of water passing through nanoscale slits, Δ*P* denotes the transmembrane pressure drop, in Pa, and *L* (nm) stands for the length of the nanoscale slit. The viscosity (*μ*) of water is 0.729 mPa·s, and *d_eff_* (nm)is the effective width of the GO slit, in nm. It is important to note that edge functional groups within the NGO slit pores usually occupy specific volumes, and thus, the volume occupied by these functional groups must be excluded when calculating the effective width of the slit. Herein, the Poreblazer code [63] was used to approximately calculate the effective width ($d_{eff}$) of the GO membrane slits. In this theoretical equation, the fitting parameter (*α*) was introduced to characterize the surface slip degree on the slit edge [62].

**Figure 5:**
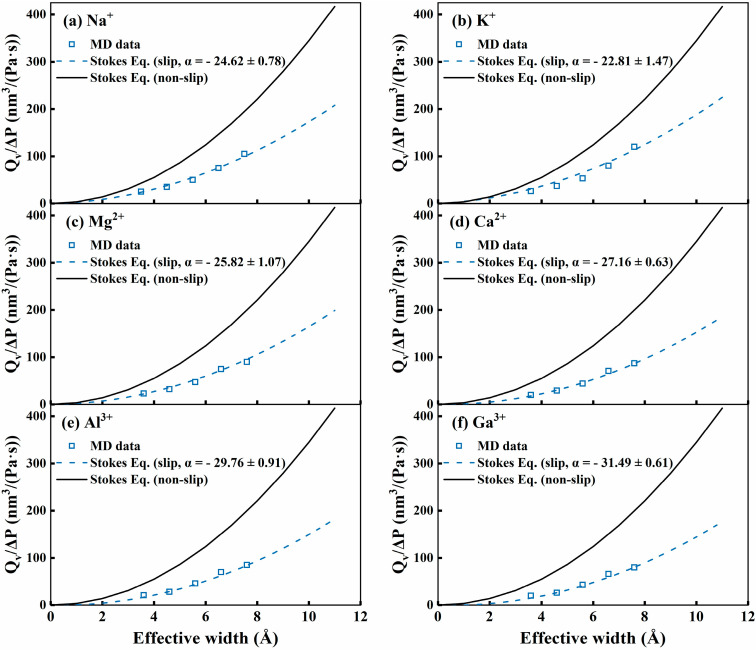
A comparison of the permeance factor (Q/∆P) of six metal ion solutions for the NGO slits between MD simulated and fitting results using the improved Stokes equation. The no-slip Stokes equation is also shown for comparison. Symbol points are the MD simulation results. The black solid lines represent the results from the no-slip Stokes equation. The blue dotted line represents the fitting results from the improved Stokes equation with the fitted parameter (α). (**a**) Na^+^; (**b**) K^+^; (**c**) Mg^2+^; (**d**) Ca^2+^; (**e**) Al^3+^; (**f**) Ga^3+^.

Figure 5 presents a comparison of water permeations between the MD simulation data and the theoretical fitting results using Equation (1) for all the simulated systems. For comparison, the original Stokes equation without consideration of surface slip [64], $\frac{Q_{v}}{∆P}=\frac{\pi L{d_{eff}}^{2}}{32\mu },$ was also used to fit the MD-based water permeation data. As shown in Figure 5, the modified Stokes equation, with the fitting parameters (*α*), can reasonably capture the simulated water permeation data in the GO slit membranes. Comparatively, a noticeable difference appears between the non-slip Stokes equation and the MD simulation data, wherein the MD simulation data is generally lower than that from the non-slip Stokes equation. This deviation indicates an important influence of edge slip phenomena on the water permeability in the GO slits. Thus, for the systems investigated in our work, the obtained negative fitting parameters (*α*) mean a negative slip process for water passing through the GO slit pores. This behavior has been extensively documented in previous reports [65,66,67,68], and it implies that there is a strong, solid–fluid interaction (adhesion) or significant surface roughness within the functionalized GO slit pores. These strong interactions or rough surfaces can lead to reduced molecular mobility near the slit edges, effectively decreasing the water-passing permeability through the slits.

It can be observed that the NGO slits usually have negative *α* values. This observation might suggest an enhanced surface interaction arising from the NGO slit pore, in which the edge -NH_2_ groups might exert a stronger interaction with water molecules. In addition, different ionic aqueous solutions also show distinctive interfacial slip behavior. That is, high-valence ions always have stronger negative slip action, imposing an impeding interaction with water molecules.

**Figure 6:**
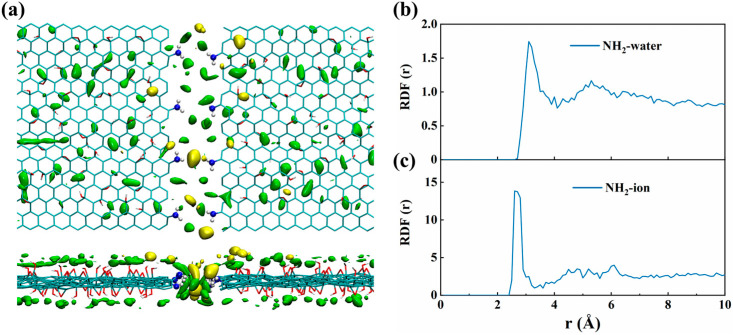
(**a**) The top and side views of the SDFs of water and ions around the NGO slit membrane. Green represents water molecules, and yellow represents metal ions. (**b**) RDFs between functional groups (-NH_2_) on the slit edges and the oxygen atoms of water molecules. (**c**) RDFs between functional groups (-NH_2_) on the slit edges and sodium ions.

Figure 6a displays the representative spatial distribution function (SDF) properties for water and ions [69] within the amine-functionalized GO slit. The SDF data can provide straightforward insight into how these edge modifications influence the packed distributions of water and cations inside the GO slits. It can be observed that water molecules are uniformly distributed within the slit pores. In addition, the cations exhibit a clustering behavior near the amine groups within the NGO slit. In order to compare the interaction strengths arising from the two functional groups, we characterized the radial distribution functions (RDFs) of water molecules and cations around the mass centers of the edge functional groups.

As shown in Figure 6b,c, the amine group can induce a higher RDF peak value. This provides support that edge amine groups impose stronger interaction with surrounding water molecules and cations, as shown above. In short, amine groups have a significant function in capturing water molecules and immobilizing cations, thus enhancing ion retention efficiency. Therefore, amine-functionalized GO slit membranes always exhibit higher ability in ion retention.

**Figure 7:**
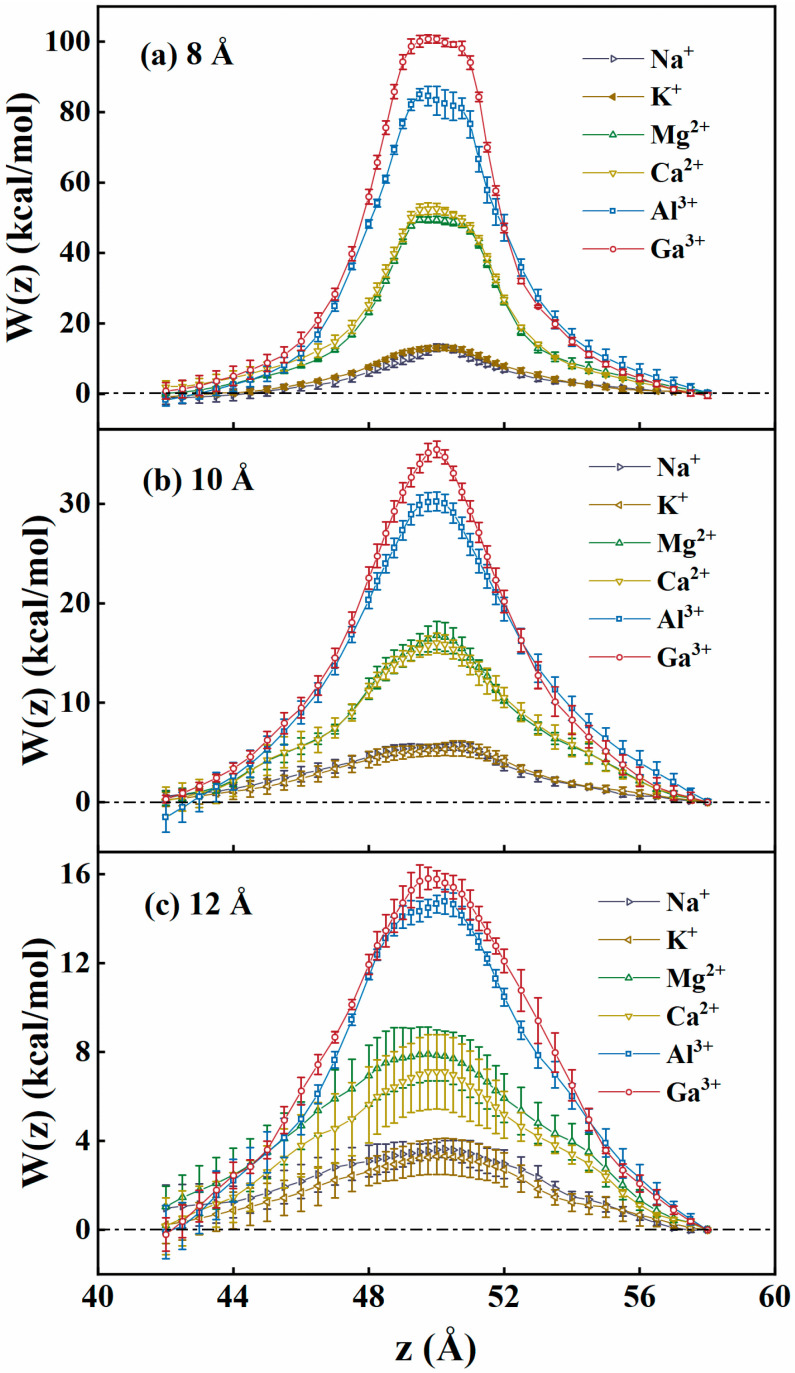
PMF profiles (W(z)) with the error bars for six metal ions crossing NGO slits along the z-direction at the slit spacings of (**a**) 8 Å, (**b**) 10 Å, and (**c**) 12 Å.

To explore the ion rejection mechanism, we simulated the potential of the mean force (PMF) profiles, *W*(z), for ions passing through the GO slits using the following integration equation [70]:

(2) $$W\left(z\right)=W\left(z_{0}\right)-\int_{z_{0}}^{z}f_{c}(z)dz$$

where $f_{c}(z)$ is the constraint force acting on the ion. $W\left(z_{0}\right)$ represents the PMF reference value with the target ion located in the bulk phase. The PMF profiles were obtained by pulling ions from one bulk phase region, through the slit center (at *z* = 50 Å), to another bulk phase region. Each PMF window was simulated for at least 6 ns to ensure that the system reached equilibrium. The pulling distance far from the positions of the NGO slit was set to 0.5 Å, while near the slit it was set to 0.25 Å to more accurately present the PMF profiles. The restraint constant was set to 1000.0 kcal/(mol·Å^2^). Figure 7 presents the PMF profiles for six types of cations through the GO slits under the slit widths of 8, 10, and 12 Å. The error bars are also shown in the figures, which were obtained by averaging runs. There are free-energy barriers for ions passing through the slit pores. The PMF profiles are almost symmetrical around the slit center for the ions in both types of GO slit membranes. The PMF barriers decrease as the slit width increases for NGO slit membranes. Generally, higher valence ions encounter larger free-energy barriers, as compared with lower valence ions. This implies that electrostatic interaction might be the critical factor determining the thermodynamic free-energy barriers.

**Figure 8:**
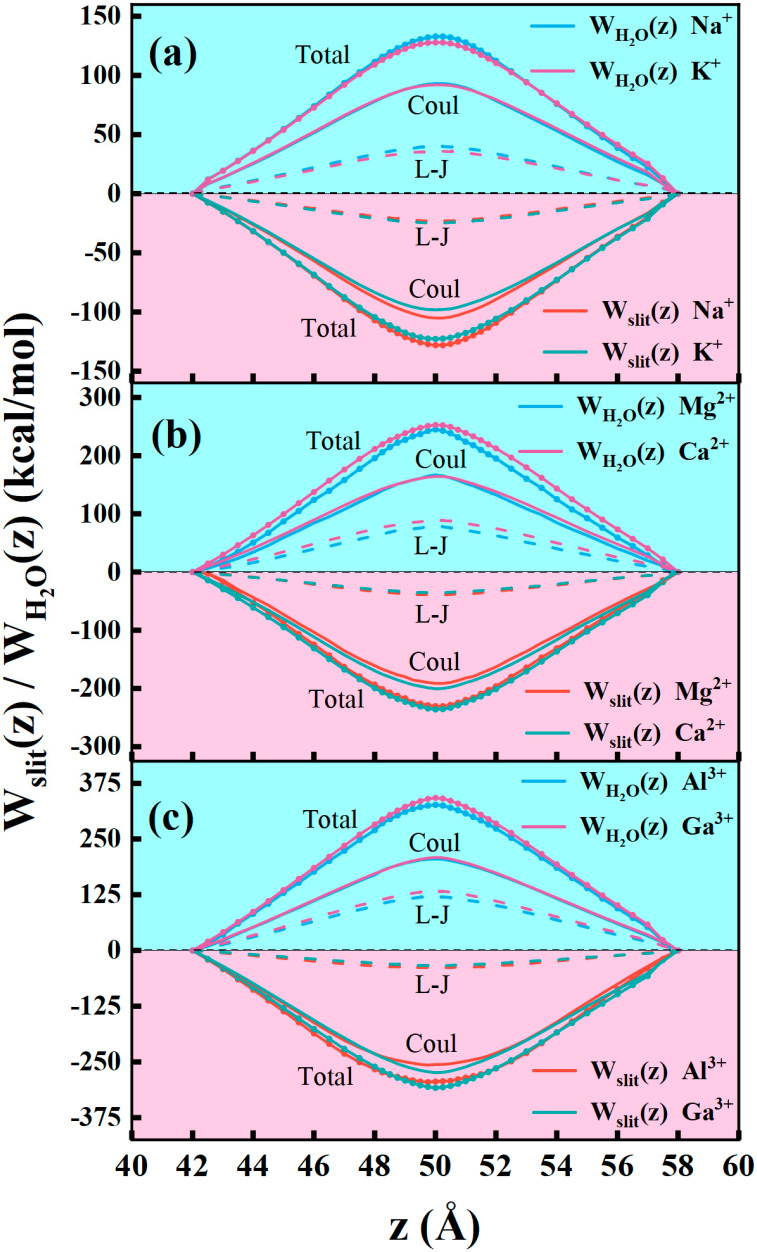
Decomposition of the PMF curves into the contributions from both the GO slits ($W_{slit}(z)$) and the water solvent ($W_{H_{2}O}(z)$) for (**a**) Na^+^ and K^+^, (**b**) Mg^2+^ and Ca^2+^, and (**c**) Al^3+^ and Ga^3+^ through the NGO slit at the width of 10 Å. The solvent contribution and the GO slit contribution are further decomposed into the L-J interaction terms and the Coulomb interaction terms.

Because the ion constraining force $f_{c}$ is exerted by the water interaction ($f_{H_{2}O}$) and the slit interaction ($f_{silt}$), that is, $f_{c}=f_{silt}+f_{H_{2}O}$. The total PMF profile was further decomposed into the interaction contributions from both the GO slits contribution (the slit term, $W_{slit}(z)$) and the water solvent contribution (the water term, $W_{H_{2}O}(z)$) [71]. Figure 8 displays the typical decomposed PMF results for six types of ions through the NGO slit membranes at the slit width (10 Å). Figures S1 and S2, respectively, show similar decomposition results for the 8 Å and 12 Å slits. These decompositions could reveal the origins of the free-energy barriers that ions encounter when they traverse the GO slits. As shown in Figure 8, the water-inducing interaction term, as an energetic barrier, generally accounts for a positive/repulsive contribution to the total PMF. The GO slit-generating interaction provides a negative/attractive role as an energy well. This suggests that the water contribution $W_{H_{2}O}(z)$ is the controlling portion in governing the PMF profile. In both the slit-generating ($W_{slit}(z)$) and the water-generating ($W_{H_{2}O}(z)$) contributions, the electrostatic interactions always play a main role in regulating the $W_{slit}(z)$ and $W_{H_{2}O}(z)$ contributions, reflecting the charged nature of ions.

**Figure 9:**
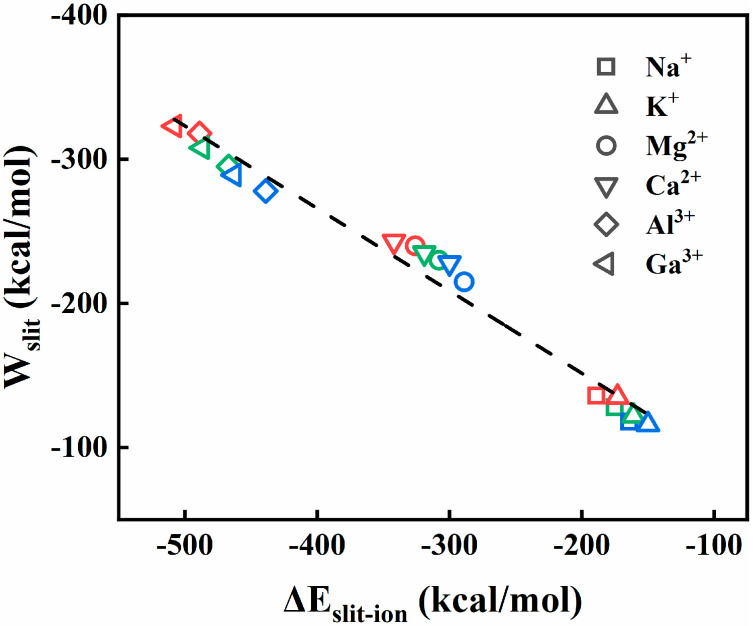
Free-energy well of the decomposed slit contribution terms ($W_{slit}$) as a function of the maximum slit–ion interaction energy $\left(∆E_{slit-ion}\right)$ for six metal ionic aqueous solutions. Three types of symbol points in different colors correspond to 8, 10, and 12 Å slit gaps.

For characterizing the properties of the slits and water contributions, the negative energetic well values ($W_{slit}$) of the decomposed slit contribution terms were plotted against the maximum slit–ion interaction energies ($∆E_{slit-ion}$) for various ionic aqueous systems. As shown in Figure 9, a linear correlation appears between the $W_{slit}$ and the $∆E_{slit-ion}$, displaying that a stronger ion–water interaction energy $∆E_{slit-ion}$ is always accompanied by an enhanced free-energy well ($W_{slit}$). This suggests that the slit contribution term is mainly determined by the interaction energy from the slit membranes.

**Figure 10:**
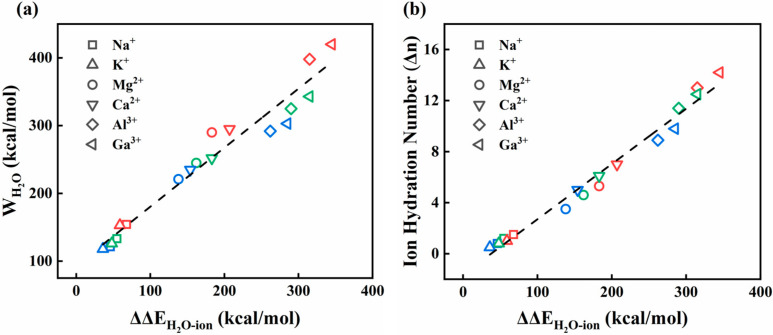
(**a**) The free-energy barriers ($W_{H_{2}O}$) and (**b**) the change in the outermost hydration numbers (∆n) of ions crossing the slits are plotted as a function of the difference between the interaction energies of ions and water in the bulk phase and the slit ($∆∆E_{H_{2}O-ion}$). Three types of symbol points in different colors correspond to 8, 10, and 12 Å gaps.

We then examine the decomposed water term $W_{H_{2}O}(z)$, which is the repulsive (positive) contribution to the total PMF. Figure 10a displays the relationship between the maximum free-energy barrier ($W_{H_{2}O}$) in the decomposed water term and the change in the ion–water interaction energy ($∆{∆E}_{H_{2}O-ion}$) from the bulk phase to the slit position, which is defined as, $∆{∆E}_{H_{2}O-ion}=∆E_{H_{2}0-ion}(slit)-∆E_{H_{2}0-ion}(bulk)$. The positive value of $∆∆E_{H_{2}O-ion}$ means a decrease in the interaction strength between the ion and surrounding water molecules when transferring from the bulk phase to the slit pore. It is also observed that there is a linear relationship between the energy barrier, $W_{H_{2}O}$, and the $∆∆E_{H_{2}O-ion}$. This means that when an ion passes through the slit, a high barrier value in the water contribution term is usually associated with a large decrease in the ion hydration energy. In addition, it is not unexpected to observe that ions with high valence exhibit an obvious reduction in the hydration energy, and thus a higher $W_{H_{2}O}\;$barrier. This linear relationship further confirms the significant contribution of ion hydration to the PMF profile during ion migration. Therefore, the thermodynamic barrier for ions passing through the edge-functionalized GO slits can be considered as the delicate combination of the ion hydration change and the slit-generated attraction.

The reduction in ion hydration interaction energy ($∆∆E_{H_{2}O-ion}$), when passing through the slit, can be attributed to the reduction in the ion hydration number, which directly affects the interaction strength between the ion and the surrounding water molecules. The ion hydration coordination number was calculated based on the number of water molecules within the cation hydration shell. The corresponding ion–water RDFs (Figure S3) were used to define the cutoff radii of the hydration shell. Herein, the first hydration shell layer, the second hydration shell layer, and the third hydration shell layer were, respectively, used for monovalent, divalent, and trivalent ions. Figure S4 shows the relevant changes in the hydration number of the ions as they pass through the 8 Å, 10 Å, and 12 Å slits. It can be found that inside the slits, there is a reduction in the ion hydration number, which represents the slit-confining effect.

Figure 10b shows the linear relationship between the change in the ion hydration energy ($∆∆E_{H_{2}O-ion}$) and the change in the ion hydration number (∆n) as the ion passes through the slit. A larger reduction in the hydration number always leads to a significant decrease in the ion hydration energy. This behavior provides support that the change in the ion hydration energy is related to the change in the ion hydration number. When the ions move from the bulk phase environment to the confined slit space, they may be forced to reduce the hydration shell due to spatial constraints within the slit pores. This reduction in hydration shells needs to overcome the hydration energy and thus makes it more difficult for the cation to pass through the slit [9]. This result further confirms the fact that the high hydrophilicity of the amine functional groups could impose strong binding stabilization toward water molecules, thus destroying the ion hydration structure within the slit.

## 4. Conclusions

In this study, we have conducted a comprehensive simulation study on the desalination performance of monolayer GO slit membranes with edge amine functionalization, e.g., edge-amino functionalized GO (NGO). Six types of ions (K⁺, Na⁺, Ca^2^⁺, Mg^2^⁺, Al^3^⁺, Ga^3^⁺) were considered in the feeding ionic solution systems. The water flux and ion rejection passing through the GO-based nanoslits were simulated for the above ionic solutions. It is found that the permeability of the NGO slit membrane shows comparable water permeability to the other slit membranes. The NGO slits can achieve the higher ion retention rates for all the cations, reflecting high ion rejection ability. The water permeation behavior through the slit pores has been analyzed by the improved Stokes equation. Our study explores a novel amine-functionalized GO membrane by incorporating neutral amine groups at the edges of GO slits. These neutral amine groups provide a unique function in capturing water molecules and immobilizing cations, thus reducing water permeation and enhancing ion retention efficiency in the NGO slit membranes. The amine-functionalized GO shows strong potential as a new type of GO membrane for desalination applications.

The underlying mechanism of the ion rejection has been analyzed by the PMF profiles for ions passing through the GO nanoslits. The GO slit-generating interaction provides an attractive role, while the ion-hydration interaction term accounts for a repulsive contribution, which is the controlling portion in governing the PMF profile. The thermodynamic barrier for ion passing can be considered as the delicate combination of the ion hydration change and the slit-generated attraction. In short, the desalination performance and the ion-rejection mechanism revealed in this work provide new functions and roles of edge neutral -NH_2_ groups, which offer design guides for developing new types of GO laminar membranes.

## Data Availability

The original contributions presented in this study are included in the article and supplementary material. Further inquiries can be directed to the corresponding author.

## Supplementary Materials

The following supporting information can be downloaded at: https://www.mdpi.com/article/10.3390/membranes15110334/s1, Figure S1: Decomposition of the PMF curves into the contribution from both the GO slits ($W_{slit}(z)$) and the water solvent ($W_{H_{2}O}(z)$) for (a) Na^+^ and K^+^, (b) Mg^2+^ and Ca^2+^, and (c) Al^3+^ and Ga^3+^ through the functionalized GO slits at the slit width of 8 Å. The solvent contribution and GO slits contribution are further decomposed into the L-J interaction terms and the Coulomb interaction terms; Figure S2: Decomposition of the PMF curves into the contribution from both the GO slits ($W_{slit}(z)$) and the water solvent ($W_{H_{2}O}(z)$) for (a) Na^+^ and K^+^, (b) Mg^2+^ and Ca^2+^, and (c) Al^3+^ and Ga^3+^ through the functionalized GO slits at the slit width of 12 Å. The solvent contribution and GO slits contribution are further decomposed into the L-J interaction terms and the Coulomb interaction terms; Figure S3: The radial distribution function (RDFs) between the six ions and oxygen atoms in water molecules (O_w_). (a) Na^+^; (b) K^+^; (c) Mg^2+^; (d) Ca^2+^; (e) Al^3+^; (f) Ga^3+^. The cutoff radii parameters corresponding to the ion hydration shell are shown. Figure S4: The profiles of the outermost hydration number for six metal ions passing through slits with the slit spacing of 8 Å, 10 Å, and 12 Å. Z = 50 represents the position of the NGO membranes; Table S1: The Tersoff–Brenner potential parameters used in C atoms on the GO sheet; Table S2: The force field parameters used in the MD simulations.
