# Supplementary material for: Microscopic Exploration of Water Permeation and Ion Rejection for Edge Amine-Functionalized GO Nanoslits
Source: Membranes (Basel). 2025 Nov 4;15(11):334. doi: 10.3390/membranes15110334 (PMC12654262; doi:10.3390/membranes15110334)
Supplement: Supplementary file 1 [file membranes-15-00334-s001.zip › membranes-3914563-supplementary.pdf]

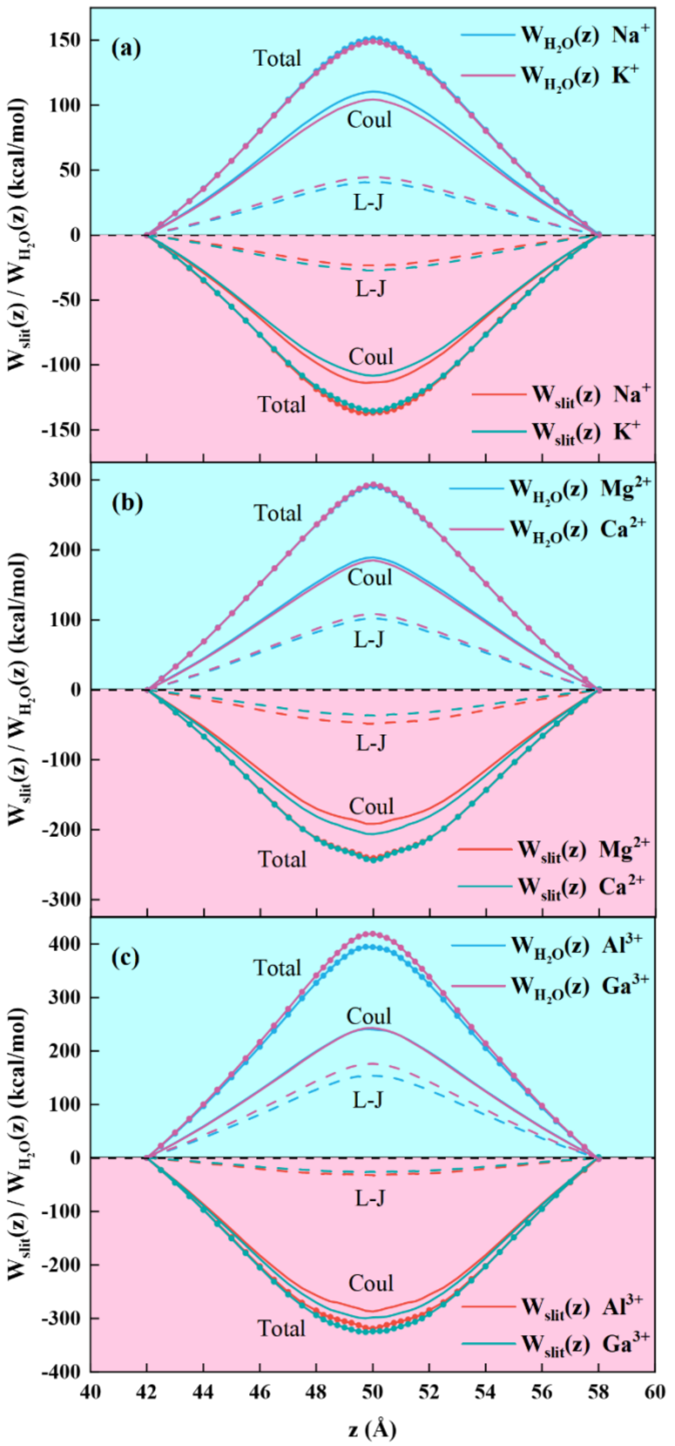

Figure S1. Decomposition of the PMF curves into the contribution from both the GO slits ($W_{slit}(z)$) and the water solvent ($W_{H_2O}(z)$) for (a) Na^+ and K^+, (b) Mg^{2+} and Ca^{2+}, and (c) Al^{3+} and Ga^{3+} through the functionalized GO slits at the slit width of 8 Å. The solvent contribution and GO slits contribution are further decomposed into the L-J interaction terms and the Coulomb interaction terms.

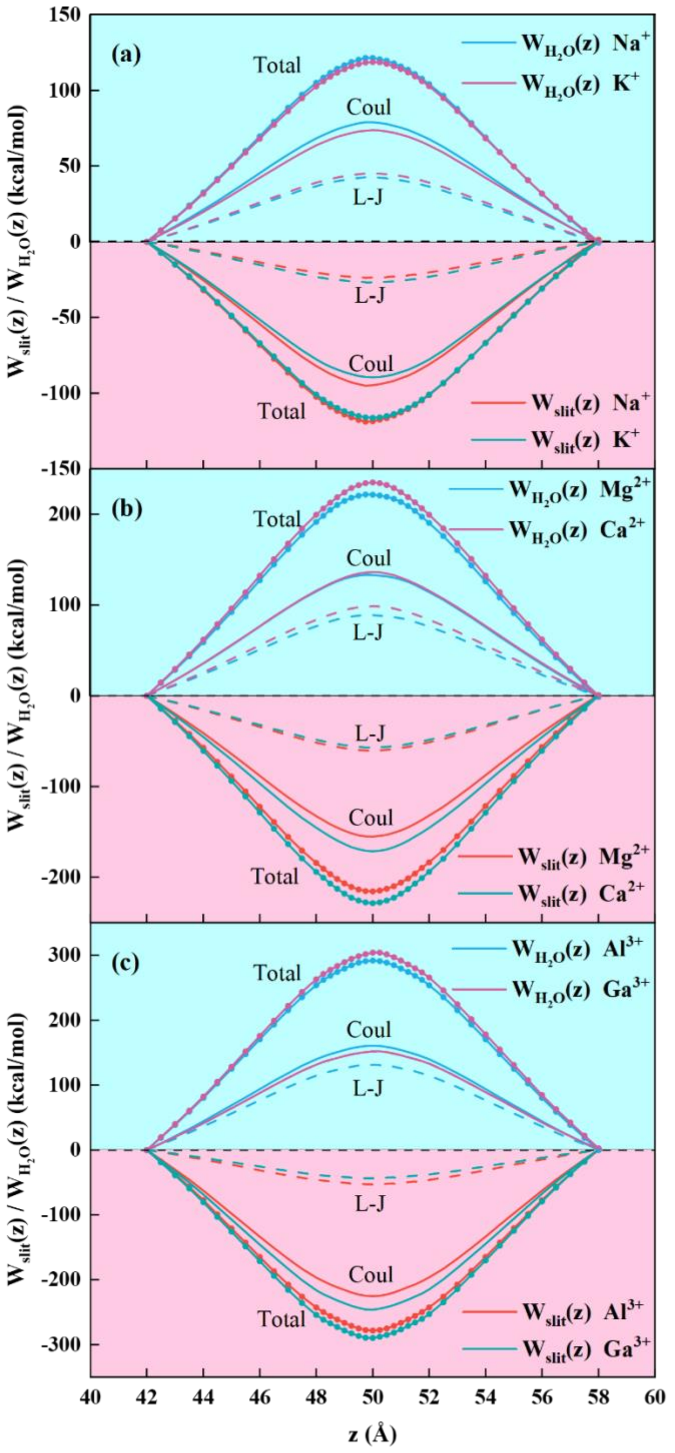

Figure S2. Decomposition of the PMF curves into the contribution from both the GO slits ($W_{slit}(z)$) and the water solvent ($W_{H_2O}(z)$) for (a) Na^+ and K^+, (b) Mg^{2+} and Ca^{2+}, and (c) Al^{3+} and Ga^{3+} through the functionalized GO slits at the slit width of 12 Å. The solvent contribution and GO slits contribution are further decomposed into the L-J interaction terms and the Coulomb interaction terms.

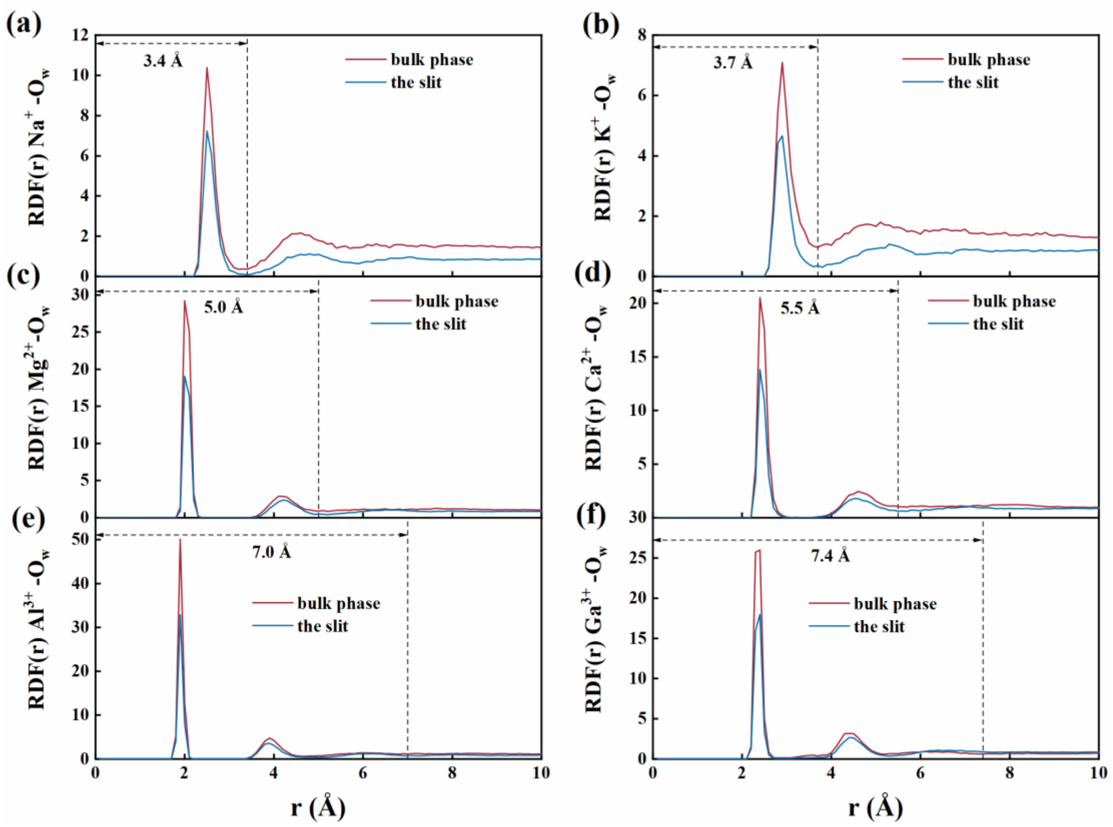

Figure S3. The radial distribution function (RDFs) between the six ions and oxygen atoms in water molecules (O_w). (a) Na^+; (b) K^+; (c) Mg^{2+}; (d) Ca^{2+}; (e) Al^{3+}; (f) Ga^{3+}. The cutoff radii parameters corresponding to the ion hydration shell are shown.

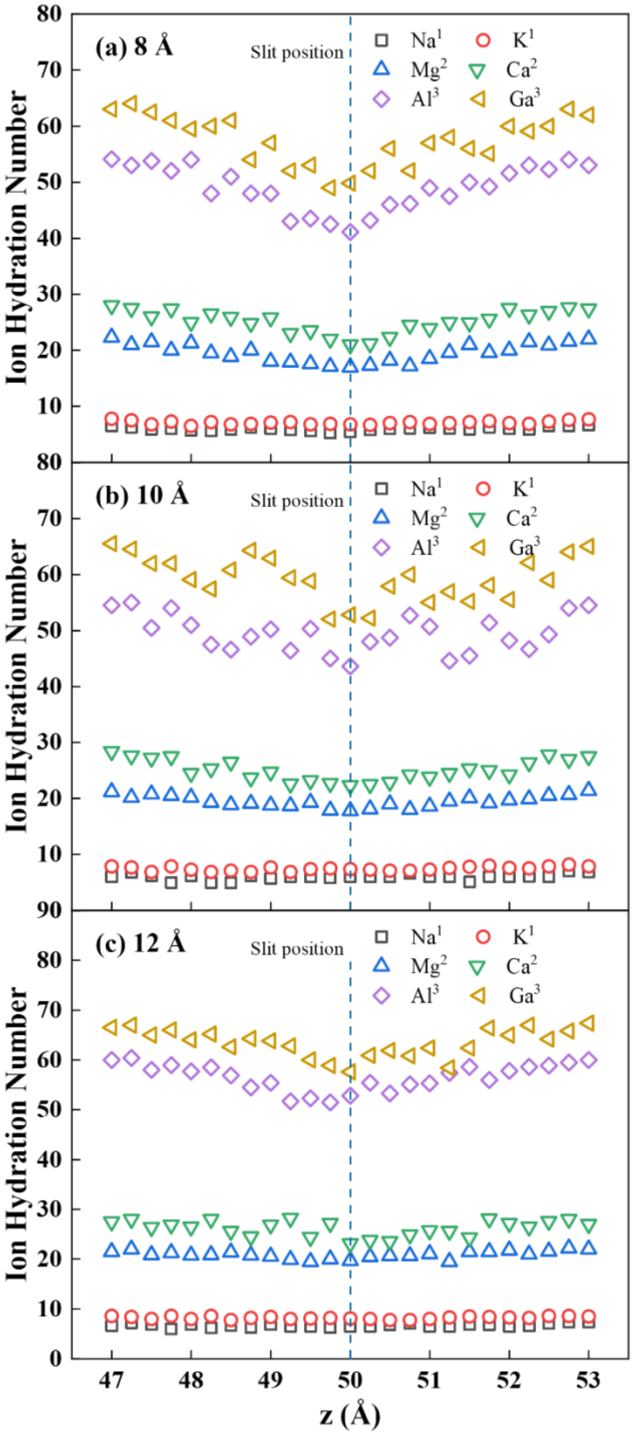

Figure S4. The profiles of the outermost hydration number for six metal ions passing through slits with the slit spacing of 8 Å, 10 Å, and 12 Å. Z = 50 represents the position of the NGO membranes.

Table S1. The Tersoff-Brenner potential parameters [1] used in C atoms on the GO sheet.

T-B	C
***A* (eV)**	1.3936×10^{3}
***B* (eV)**	3.467×10^{2}
***λ* (Å^{-1})**	3.4879
***μ* (Å^{-1})**	2.2119
β	1.5724×10^{-7}
n	7.2751×10^{-1}
c	3.8049×10^{4}
d	4.384×10^{0}
h	-5.7058×10^{-1}
***R* (Å^{-1})**	1.8
***S* (Å^{-1})**	2.1

In the simulations, the sum of Lennard-Jones (LJ) and Coulomb interactions, as expressed in the Equation 1, was used to describe the non-bonded intetraction.

$$V_{LJ} = 4\varepsilon_{ij}\left[\left(\frac{\sigma_{ij}}{r_{ij}}\right)^{12} - \left(\frac{\sigma_{ij}}{r_{ij}}\right)^{6}\right] + \frac{q_i q_j}{r_{ij}} \quad (1)$$

where ε_{ij} and σ_{ij} are the LJ energy and size parameters, r_{ij} is the distance between atoms i and j, and q_i, q_j are the atomic partial charges.

The bond and angle interactions were described using harmonic potentials, as shown in the following Equations:

$$V_{bond}(r) = k_b(r - r_0)^2 \quad (2)$$

where r_0 are the equilibrium bond length, and k_b are the corresponding force constants.

$$V_{angle}(\theta) = k_\theta(\theta - \theta_0)^2 \quad (3)$$

where r_0 are the equilibrium angle length, and k_b are the corresponding force constants.

Dihedral interactions were described using opls potentials, as shown in Equation 4:

$$V_{dihedral}(\emptyset) = \frac{1}{2}[K_1(1 + cos\emptyset) + K_2(1 - cos2\emptyset) + K_3(1 + cos3\emptyset) + K_1(1 - cos4\emptyset)] \quad (4)$$

where $\emptyset$ is the dihedral angle, and K_1 to K_4 are the Fourier coefficients specific to each dihedral type.

Table S2. The force field parameters used in the MD simulations.

Atoms	ε (kcal/mol)	σ (Å)	q (e)	References
**C(C-C)**	0.0557	3.3997	0	[1]
**O(C-OH)**	0.17	3.07	-0.585	[2-4]
**H(C-OH)**	0.00	0.00	+0.435	[2-4]
**O(C-O)**	0.14	2.90	-0.28	[2-4]
**N(C-NH$_2$)**	0.276	3.50	-0.9768	[2-4]
**H(C-NH$_2$)**	0.00	0.00	+0.4884	[2-4]
**O$_W$**	0.1553	3.166	-0.8476	[5]
**H$_W$**	0.00	0.00	+0.4238	[5]
**Na^+**	0.1046	2.586	+1.0	[6, 7]
**K^+**	0.1046	3.331	+1.0	[6, 7]
**Mg^{2+}**	0.9153	1.398	+2.0	[8]
**Ca^{2+}**	0.4704	2.361	+2.0	[8]
**Al^{3+}**	0.2166	1.4472	+3.0	[9, 10]
**Ga^{3+}**	1.0642	2.1738	+3.0	[10]
**Cl^-**	0.1	4.4	-1.0	[6-8]

Bonds	r_0/ Å	k_r/(kcal mol^{-1} Å^{-2})
**C-O(C-OH)**	1.41	320
**O=H(C-OH)**	0.945	553
**C-O(C-O)**	1.41	320
**C-N(C-NH$_2$)**	1.44	319.7
**N-H(C-NH$_2$)**	1.01	363.2
**O$_W$-H$_W$**	1.0	450

Angles	θ/deg	kθ/(kcal mol^{-1} rad^{-2})
**C-O-C(C-O)**	63.9	60
**C-O-H(C-OH)**	108.5	55
**C-C-N**	110	29.3
**C-N-H**	105.9	36.5
**H$_W$-O$_W$-H$_W$**	109.47	55.0

Dihedrals	K_1/(kcal/mol)	K_2	K_3	K_4
**C-C-N-H**	-0.1900	-0.4171	0.4180	0.00

Reference

[1] J. Tersoff, Modeling solid-state chemistry: Interatomic potentials for multicomponent systems, Physical Review B, 39 (1989) 5566-5568.

[2] W.L. Jorgensen, D.S. Maxwell, J. TiradoRives, Development and testing of the OPLS all-atom force field on conformational energetics and properties of organic liquids, J. Am. Chem. Soc., 118 (1996) 11225-11236.

[3] W. Damm, A. Frontera, J. Tirado-Rives, W.L. Jorgensen, OPLS all-atom force field for carbohydrates, J. Comput. Chem., 18 (1997) 1955-1970.

[4] W.D. Cornell, P. Cieplak, C.I. Bayly, I.R. Gould, K.M. Merz, D.M. Ferguson, D.C. Spellmeyer, T. Fox, J.W. Caldwell, P.A. Kollman, A second generation force field for the simulation of proteins, nucleic acids, and organic molecules, J. Am. Chem. Soc., 117 (1995) 5179-5197.

[5] H.J.C. Berendsen, J.R. Grigera, T.P. Straatsma, The missing term in effective pair potentials, J. Phys. Chem., 91 (1987) 6269-6271.

[6] L. X. Dang, Mechanism and thermodynamics of ion selectivity in aqueous-solutions of 18-crown-6 ether: a molecular dynamics study, J. Am. Chem. Soc., 117 (1995) 6954-6960.

[7] S. Chowdhuri, A. Chandra, Hydration structure and diffusion of ions in supercooled water: ion size effects, J. Chem. Phys., 118 (2003) 9719-9725.

[8] J.P. Larentzos, L.J. Criscenti, A molecular dynamics study of alkaline earth metal-chloride complexation in aqueous solution, J. Phys. Chem. B, 112 (2008) 14243-14250.

[9] T.M.C. Faro, G.P. Thim, M.S. Skaf, A Lennard-Jones plus Coulomb potential for Al3+ ions in aqueous solutions, J. Chem. Phys., 132 (2010) 114509.

[10] T. Kowall, P. Caravan, H. Bourgeois, L. Helm, F.P. Rotzinger, A.E. Merbach, Interpretation of activation volumes for water exchange reactions revisited: Ab initio calculations for Al3+, Ga3+, and In3+, and new experimental data, J. Am. Chem. Soc., 120 (1998) 6569-6577.
